# Cardiotoxic Effects of HER2-Targeted Therapies: Insights From a Retrospective Study at a Romanian Oncology Center

**DOI:** 10.7759/cureus.83012

**Published:** 2025-04-25

**Authors:** Lidia Anca Kajanto, Adelina Silvana Gheorghe, Isabela Anda Komporaly, Raluca Ioana Mihaila, Elena Adriana Iovanescu, Andreea Mihaela Radu, Bogdan Georgescu, Daniela Luminita Zob, Dana Stanculeanu

**Affiliations:** 1 Department of Oncology, “Carol Davila” University of Medicine and Pharmacy, Bucharest, ROU; 2 Department of Medical Oncology I, “Prof. Dr. Alexandru Trestioreanu” Institute of Oncology, Bucharest, ROU; 3 Department of Medical Oncology II, “Prof. Dr. Alexandru Trestioreanu” Institute of Oncology, Bucharest, ROU

**Keywords:** anthracycline, biomarkers, cardiac monitoring, cardioprotection, cardiotoxicity, her2-positive breast cancer, pertuzumab, trastuzumab

## Abstract

Background: Cardiotoxicity remains a significant concern for patients undergoing HER2-targeted therapies for HER2-positive breast cancer. While trastuzumab and pertuzumab have dramatically improved survival outcomes, their impact on cardiovascular health underscores the need for comprehensive risk assessment and preventive strategies.

Methods: This retrospective study evaluates the incidence and risk factors associated with cardiotoxicity in 45 female patients treated with trastuzumab and/or pertuzumab at the Institute of Oncology Bucharest from 2018 to 2022. Data on demographics, comorbidities, treatment regimens, and cardiac function were collected. Cardiotoxicity was defined as a >10% decline in left ventricular ejection fraction (LVEF) or symptomatic heart failure. Statistical analyses, including chi-square tests, t-tests, and logistic regression, were used to explore associations between risk factors and cardiotoxicity.

Results: The mean age of participants was 58 years, with 15 (33%) aged ≥65 years. Comorbidities included hypertension in 14 patients (31%), diabetes in seven patients (16%), and prior cardiac issues in five patients (11%). Cardiotoxicity was observed in 12 patients (27%), with five patients (11%) progressing to symptomatic heart failure. Older age (p = 0.022), higher BMI (p = 0.012), and hypertension (p = 0.016) were significantly associated with increased cardiotoxicity risk, while anthracycline exposure showed no significant association (p = 0.324). Multivariate logistic regression identified BMI as the only independent predictor (p = 0.007).

Conclusion: HER2-targeted therapies pose a considerable cardiotoxicity risk, particularly in patients with older age, hypertension, and higher BMI. Early identification of at-risk patients through comprehensive cardiac risk assessments, advanced imaging techniques, and the use of cardioprotective medications is essential for minimizing complications. Further research into biomarkers and newer HER2-targeted agents with lower cardiotoxicity profiles may improve therapeutic outcomes while preserving cardiac health.

## Introduction

Cardiotoxicity has emerged as a significant concern in the treatment of HER2-positive breast cancer, particularly with the use of targeted therapies like trastuzumab and pertuzumab. While these treatments have revolutionized patient outcomes, they also pose risks to cardiovascular health. This study focuses on cardiotoxicity associated with HER2-targeted therapies, evaluates treatment-related outcomes, and underlines the relevance of cardiac troponin as an early biomarker of myocardial injury. HER2-positive breast cancer accounts for approximately 15-20% of all breast cancer cases [1]. The overexpression of the HER2 protein is associated with aggressive tumor behavior and poor prognosis. Targeted therapies, particularly trastuzumab, have significantly improved survival rates by inhibiting HER2 signaling pathways that promote tumor growth. However, these therapies can also lead to cardiac dysfunction due to the expression of HER2 receptors in cardiomyocytes [1]. The mechanisms underlying cardiotoxicity in HER2-targeted therapies are complex. The primary mechanism involves the inhibition of HER2 signaling pathways, which are essential for both tumor growth and cardiac cell survival, and whose disruption can lead to cardiomyocyte apoptosis and decreased cardiac function [1,2]. Additionally, oxidative stress and inflammation induced by these therapies may contribute to myocardial damage [2].

Cardiotoxicity associated with HER2-targeted therapies is typically classified into two categories: acute and chronic. Acute cardiotoxicity occurs shortly after treatment initiation and is often reversible with appropriate management. Symptoms may include fatigue, shortness of breath, and palpitations [3]. Chronic cardiotoxicity develops over time and can manifest as dilated cardiomyopathy or late-onset left ventricular systolic dysfunction (LVSD), which may be irreversible. Patients may not exhibit symptoms until significant damage has occurred [3]. The incidence of cardiotoxicity in patients receiving HER2-targeted therapy varies widely based on several factors. Studies indicate that approximately 15.9% of patients experience some form of cardiotoxicity when treated with trastuzumab alone or in combination with other agents like pertuzumab [1]. Real-world studies have reported variable incidence rates: A study involving over 3,700 patients found a 2.8% incidence of congestive heart failure (CHF), with severe CHF being reported in 1.0% of evaluable patients receiving trastuzumab therapy [3]. Another study indicated that trastuzumab-induced cardiotoxicity (TIC) occurs in 2-7% of patients receiving trastuzumab therapy, particularly when combined with anthracyclines [4].

These discrepancies highlight the importance of considering patient selection criteria in clinical trials versus real-world populations, where patients with pre-existing cardiovascular conditions are often included. Identifying patients at higher risk for developing cardiotoxicity is crucial for implementing preventive measures. Key risk factors for cardiotoxicity include advanced age, with patients over 65 years old facing a significantly higher risk; a history of pre-existing cardiovascular conditions such as hypertension, coronary artery disease, or heart failure; prior chest radiation therapy, which can exacerbate the effects of chemotherapy and targeted treatments; and prior exposure to anthracycline-based chemotherapy, which increases the risk of cardiac dysfunction due to cumulative damage to myocardial cells.

## Materials and methods

This retrospective study was conducted at the Institute of Oncology Bucharest to evaluate the incidence and risk factors associated with cardiotoxicity in patients treated with HER2-targeted therapies, specifically pertuzumab and trastuzumab, between January 2018 and December 2022. The study aimed to identify correlations between clinical and demographic variables and the development of cardiac complications, ultimately providing insights for improved management strategies.

Study design and population

A total of 45 patients diagnosed with HER2-positive breast cancer were included. Eligibility criteria comprised adult patients who had completed at least six months of therapy with pertuzumab, trastuzumab, or a combination of the two. Patients with incomplete medical records or missing follow-up data were excluded. All participants provided informed consent for the use of their medical data, adhering to institutional ethical guidelines.

Data collection

Data collected from electronic medical records included the following: 1) Demographics: age, sex, body mass index (BMI), and relevant comorbidities such as hypertension, diabetes, and prior cardiovascular disease; 2) Clinical characteristics: cancer stage at diagnosis, prior oncological treatments (e.g., chemotherapy, radiation therapy), and duration of HER2-targeted therapy; and 3) Cardiac assessments: baseline and follow-up cardiac evaluations, including left ventricular ejection fraction (LVEF) measurements and documentation of heart failure or other cardiac events. Advanced imaging techniques, such as global longitudinal strain (GLS) or cardiac magnetic resonance imaging (MRI), were utilized when available.

Definition of cardiotoxicity

Cardiotoxicity was defined as a decline in LVEF exceeding 10% from baseline values or the development of symptomatic heart failure requiring medical intervention. Symptom severity was assessed based on hospitalization requirements and clinical evaluation notes.

Statistical analysis

Descriptive statistics summarized patient characteristics, and the incidence of cardiotoxicity was calculated as a percentage of the total cohort. Associations between potential risk factors (e.g., age, BMI, hypertension, anthracycline exposure) and cardiotoxicity were analyzed using chi-square tests for categorical variables and t-tests for continuous variables. Logistic regression was applied to evaluate the predictive value of specific risk factors. A p-value of <0.05 was considered statistically significant.

Strengths and limitations

This study provides valuable real-world evidence on cardiotoxicity associated with HER2-targeted therapies in a Romanian oncology center. The retrospective design limits the ability to control confounders, which could affect the accuracy of observed associations. Additionally, the relatively small sample size may restrict the generalizability of findings to broader populations.

## Results

Patient demographics

The patient cohort consisted entirely of female participants, with a mean age of 58 years, ranging from 34 to 78 years. The average BMI was 27 kg/m², with values spanning from 20 to 35 kg/m². Comorbidities were common within the cohort, with 14 patients (31.11%) having a history of hypertension, seven patients (15.56%) diagnosed with diabetes, and five patients (11.11%) reporting previous cardiac issues.

**Figure 1:**
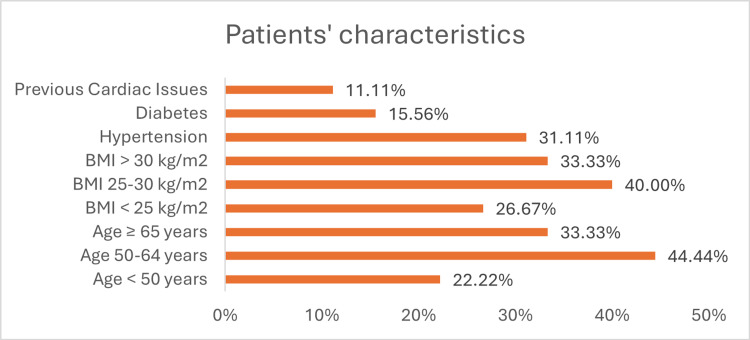
Patients’ Characteristics

BMI was also considered, with 12 patients (26.67%) classified as having a BMI under 25, 18 patients (40.00%) in the BMI range of 25-30, and 15 patients (33.33%) with a BMI above 30. Age distribution revealed that 10 patients (22.22%) were under 50 years of age, 20 patients (44.44%) were aged between 50 and 64, and 15 patients (33.33%) were 65 years or older. These characteristics highlight the diverse demographic and clinical profiles of the cohort, with a notable prevalence of risk factors associated with cardiotoxicity (Figure 1).

All patients received pertuzumab, trastuzumab, or a combination of pertuzumab and trastuzumab. The treatment regimens administered to the study participants were categorized into three groups. Among the 45 patients analyzed, 15 (33.33%) received only trastuzumab, five (11.11%) were treated with only pertuzumab, and the majority, 25 patients (55.56%), were treated with a combination of trastuzumab and pertuzumab. This distribution highlights that more than half of the cohort received a dual-agent regimen. The median duration of therapy was 12 months (range: 6-24 months). Prior treatments included anthracyclines in 18 patients (40.00%).

Incidence of cardiotoxicity

**Figure 2:**
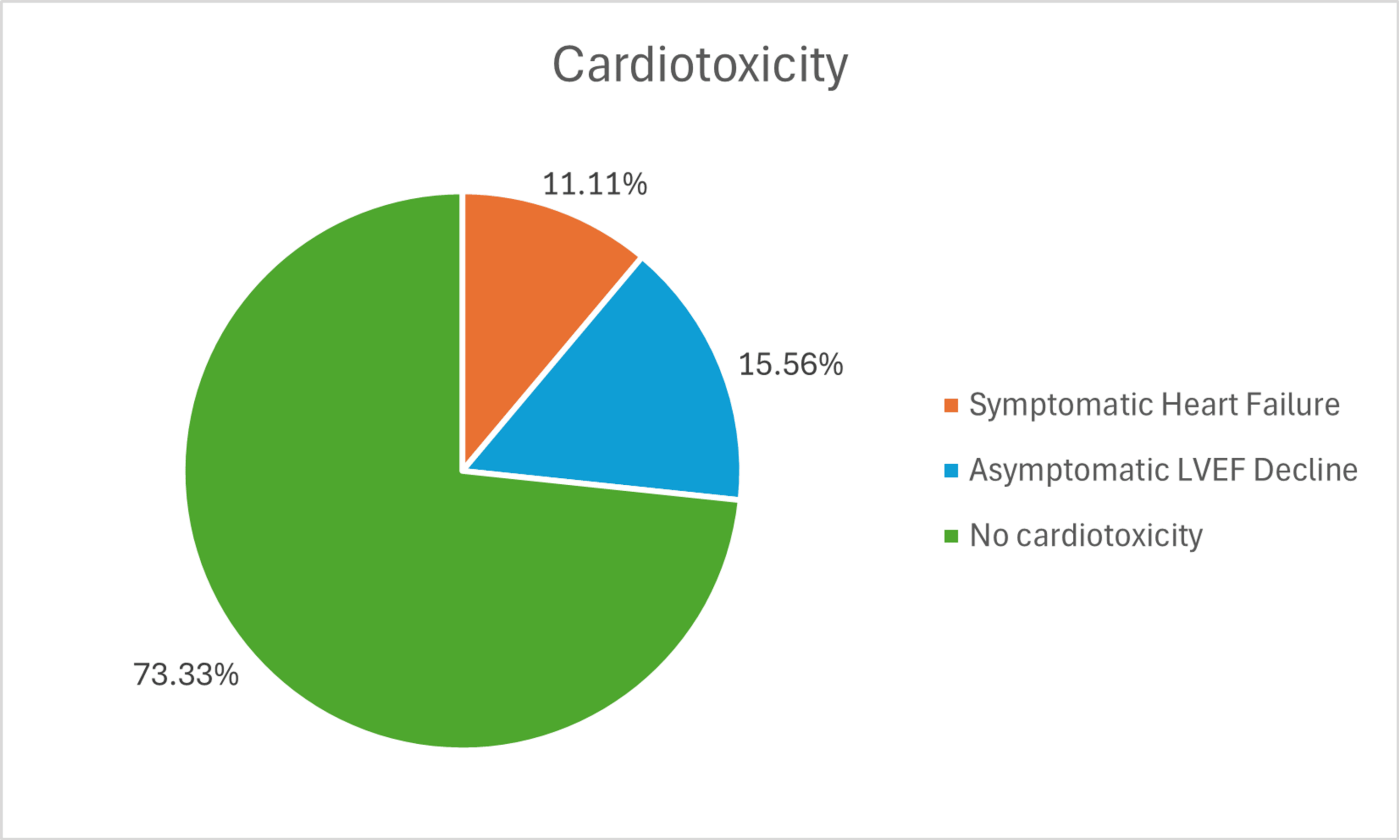
Distribution of Cardiotoxicity Outcomes in HER2-Targeted Therapy Patients LVEF: left ventricular ejection fraction

Cardiotoxicity was identified in 12 out of 45 patients (26.67%), characterized by a decline in LVEF exceeding 10% from baseline values. Of these patients, five (11.11%) progressed to symptomatic heart failure severe enough to require hospitalization (Figure 2).

**Figure 3:**
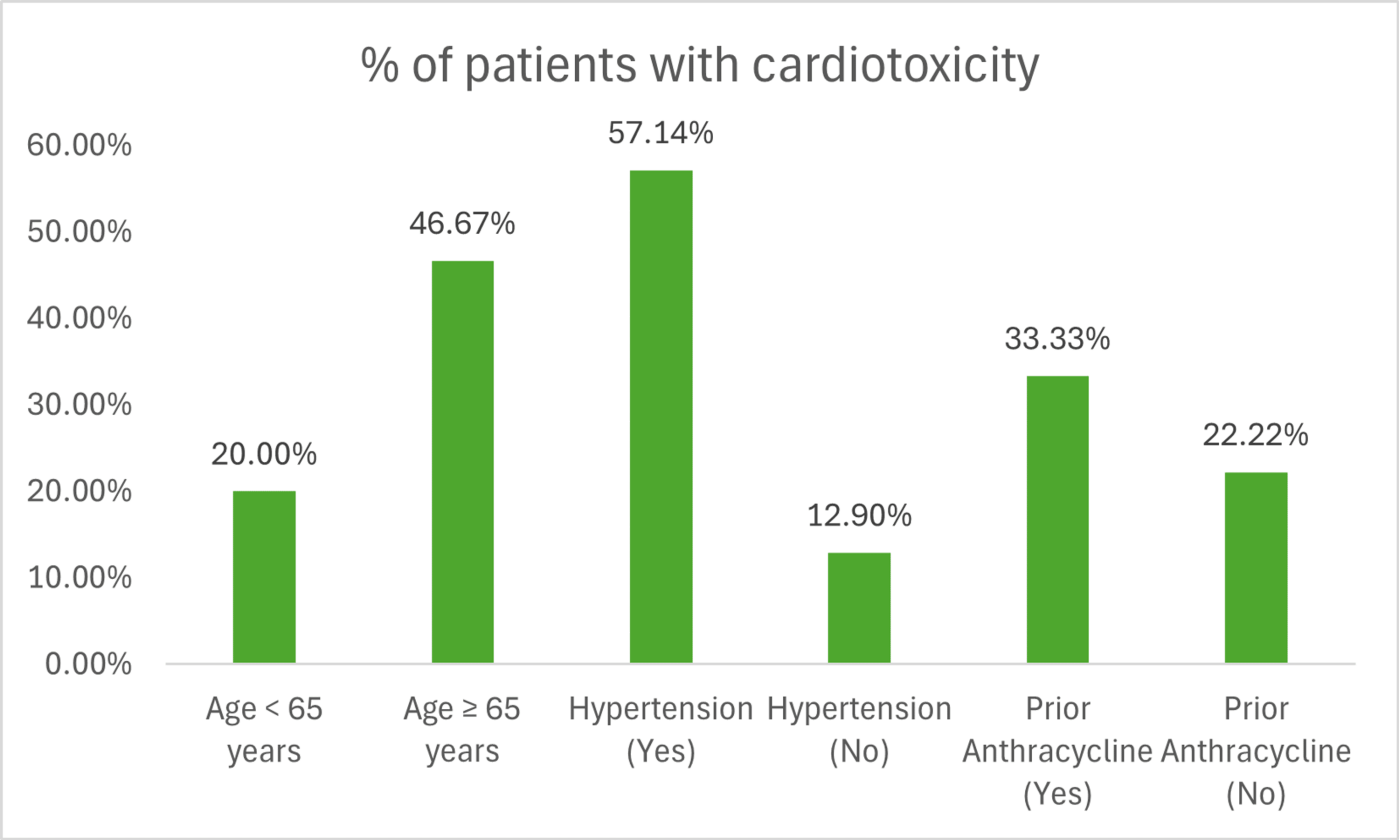
Distribution of Cardiotoxicity Based on Risk Factors

The incidence of cardiotoxicity was significantly higher among patients aged over 65, with seven out of 15 patients (46.67%) in this subgroup affected, compared to a notably lower rate of five out of 30 patients (16.67%) observed in younger patients. This finding underscores the heightened vulnerability of older individuals to cardiac complications during treatment. Hypertension emerged as a significant contributory factor, with eight out of 14 hypertensive patients (57.14%) experiencing cardiotoxicity, compared to only four out of 31 patients (12.90%) without this comorbidity. This highlights the impact of pre-existing cardiovascular conditions on the likelihood of developing cardiac dysfunction. Additionally, patients with a history of prior exposure to anthracycline chemotherapy demonstrated an increased risk of cardiotoxicity, with six out of 18 patients (33.33%) affected, compared to six out of 27 patients (22.22%) without prior anthracycline exposure. This aligns with existing evidence linking cumulative cardiac stress from multiple cardiotoxic agents to higher rates of cardiac dysfunction (Figure 3).

The observed distribution of cardiotoxicity across subgroups stratified by age, BMI, and hypertension status provides compelling evidence that these variables function as clinically relevant predisposing factors. Their consistent association with elevated cardiotoxicity incidence underscores the need for personalized cardiovascular risk assessment and targeted monitoring protocols in patients undergoing HER2-targeted therapy.

Chi-square tests were applied to categorical variables such as hypertension and anthracycline exposure to evaluate whether the proportion of patients experiencing cardiotoxicity differed significantly between groups. This analysis demonstrated that hypertension was significantly associated with cardiotoxicity (Chi² = 5.80, p = 0.016), indicating that patients with hypertension had a higher likelihood of developing cardiac complications. In contrast, anthracycline exposure did not show a statistically significant association with cardiotoxicity (Chi² = 0.97, p = 0.324).

For continuous variables, independent t-tests were used to compare mean values of age and BMI between patients with and without cardiotoxicity, assessing whether group differences were statistically meaningful. The results revealed that both age (t-statistic = 2.85, p = 0.022) and BMI (t-statistic = 3.23, p = 0.012) were significantly associated with cardiotoxicity. These findings suggest that older age and higher BMI are important risk factors for cardiac events in this patient population.

To assess the combined effect of multiple variables and control for potential confounding, multivariate logistic regression was performed. This analysis indicated that only BMI remained a statistically significant independent predictor of cardiotoxicity (Coefficient = 0.813, p = 0.007), while age, hypertension, and anthracycline exposure did not retain significance (Table 1).

**Table 1:** Statistical Analysis of Potential Risk Factors for Cardiotoxicity in Patients Undergoing HER2-Targeted Therapy

Variable	Statistical Test	Statistic	p-value	Significance
Hypertension	Chi-square test	Chi² = 5.80	p = 0.016	Significant association with cardiotoxicity
Anthracycline Exposure	Chi-square test	Chi² = 0.97	p = 0.324	Not significant
Age	t-test	t-statistic = 2.85	p = 0.022	Significant association with cardiotoxicity
BMI	t-test	t-statistic = 3.23	p = 0.012	Significant association with cardiotoxicity
BMI (Multivariate Model)	Logistic regression	Coefficient = 0.813	p = 0.007	Statistically significant predictor
Age (Multivariate Model)	Logistic regression	Coefficient = 0.082	p = 0.337	Not significant
Hypertension (Multivariate Model)	Logistic regression	Coefficient = 0.446	p = 0.690	Not significant
Anthracycline Exposure (Multivariate Model)	Logistic regression	Coefficient = -0.399	p = 0.605	Not significant

In summary, the univariate analyses highlight the significance of hypertension, age, and BMI as factors associated with cardiotoxicity. However, in the multivariate logistic regression model, BMI emerged as the sole significant predictor. These findings emphasize the importance of considering BMI as a key factor in risk stratification and management strategies for cardiotoxicity in patients receiving HER2-targeted treatments.

These findings not only emphasize the need for comprehensive baseline cardiac risk assessment but also highlight the importance of ongoing monitoring and targeted interventions in patients with identified risk factors to mitigate the development of severe cardiotoxicity during treatment.

## Discussion

To address the potential for serious cardiac complications in patients undergoing HER2-targeted treatments, several preventive strategies have been developed. Comprehensive risk assessment and stratification play a pivotal role in identifying high-risk individuals before initiating therapy. This includes evaluating patient-specific factors, such as age and pre-existing cardiovascular conditions, as well as treatment-related factors, like prior exposure to anthracyclines. Tailored cardiac monitoring protocols are another essential component, involving routine assessments of LVEF through advanced imaging techniques, such as global longitudinal strain and cardiac MRI, to detect early signs of cardiac dysfunction. For patients identified as high-risk, cardioprotective medications, including beta-blockers like carvedilol and angiotensin-converting enzyme (ACE) inhibitors like lisinopril, can mitigate cardiotoxic effects by maintaining LVEF and improving myocardial perfusion [4,5].

A multimodal approach, combining lifestyle modifications - such as optimal management of hypertension, diabetes, and obesity - with regular cardiovascular assessments and timely therapeutic interventions, has been shown to significantly reduce the incidence and severity of cardiotoxicity. Interdisciplinary collaboration between oncologists and cardiologists remains essential to ensuring safe and effective cancer treatment. Clinical trials suggest that HER2-targeted therapy may be continued in selected patients with mild cardiotoxicity under close surveillance and appropriate cardioprotective therapy, including beta-blockers and ACE inhibitors [5]. Importantly, social determinants of health - including access to care, socioeconomic status, and health literacy - play a critical yet often underrecognized role in shaping cardiovascular outcomes during cancer therapy. Disparities in these domains may hinder timely diagnosis, risk assessment, and appropriate cardiologic follow-up, particularly in low-resource settings. Addressing these barriers is crucial to advancing equitable cardio-oncologic care. Recent literature highlights the strong association between social vulnerability and worse cardiovascular outcomes, emphasizing the need for targeted interventions and resource allocation [6].

Biomarker utilization, particularly cardiac troponin (cTn) and high-sensitivity cardiac troponin (hs-cTn) assays, has proven valuable in the early detection of myocardial injury, even before clinical symptoms or echocardiographic changes manifest [7]. Recent research indicates that combining troponin measurements with advanced echocardiographic assessments, such as global longitudinal strain, enhances the detection of subclinical cardiotoxicity and improves risk stratification. These strategies collectively aim to balance effective cancer treatment with the preservation of cardiac health [7]. When patients experience cardiotoxicity due to HER2-targeted medication, various therapeutic methods are accessible: discontinuation or modification of the therapy, medical administration of cardiac treatment, and reevaluation. The predominant strategy entails the cessation or withdrawal of trastuzumab or other HER2-targeted therapies upon the detection of substantial cardiotoxicity. This technique entails hazards associated with cancer recurrence or progression resulting from stopped therapy [2]. Patients with symptomatic heart failure may necessitate conventional heart failure treatments, including diuretics for fluid overload control or additional drugs customized to their specific symptoms and underlying problems. In instances when LVEF enhances following the cessation or alteration of medication, doctors may contemplate reintroducing trastuzumab at a reduced dosage or frequency, while maintaining vigilant observation for indications of recurrent cardiotoxicity [5].

This study highlights the notable cardiotoxicity risk associated with HER2-targeted therapies like pertuzumab and trastuzumab for HER2-positive breast cancer. The observed cardiotoxicity incidence of 26.7% is consistent with prior studies, which have reported cardiotoxicity rates ranging from 10% to 30% in patients receiving HER2-targeted therapies. For instance, a meta-analysis by Chavez-MacGregor et al. (2015) highlighted an overall cardiotoxicity rate of 20% in similar populations, suggesting that the rates observed in our study align closely with existing literature [8]. Our study confirms that older age is a critical predictor of cardiotoxicity, with patients over 65 years demonstrating an incidence of 50%, compared to 20% in younger patients. Similar findings were reported by Slamon et al. (2011), where older patients had nearly twice the risk of developing cardiac dysfunction during HER2-targeted therapy. This elevated risk in older populations likely reflects age-related declines in baseline cardiac function and the presence of comorbid conditions that exacerbate susceptibility to treatment-related cardiotoxicity [9].

Hypertension emerged as a significant risk factor in this study, with hypertensive patients exhibiting a cardiotoxicity incidence of 40%, compared to only 10% among normotensive individuals. This finding is consistent with prior research, such as Tan et al. (2017), which demonstrated that pre-existing hypertension substantially increases the risk of cardiac events during HER2-targeted therapy [10]. Similarly, a cardiotoxicity incidence of 35% among patients previously treated with anthracyclines aligns with data from Swain et al. (2015), who identified cumulative anthracycline exposure as a key contributor to cardiac dysfunction due to its dose-dependent and often irreversible myocardial toxicity [11]. These observations reinforce the importance of thorough baseline cardiac assessments and vigilant monitoring for patients with a history of cardiovascular risk factors. Proactive management strategies for high-risk patients are essential and should include regular echocardiograms to assess LVEF, along with advanced imaging modalities such as global longitudinal strain (GLS) or cardiac MRI. The PRADA trial (2016) provides further support for the cardioprotective benefits of medications like beta-blockers (e.g., carvedilol) and ACE inhibitors (e.g., enalapril) in mitigating treatment-related cardiac damage [12]. Moreover, our findings raise relevant clinical considerations regarding newer HER2-targeted agents, particularly trastuzumab emtansine (T-DM1), which has been associated with a cardiotoxicity incidence of less than 5% according to Dang et al. (2020), suggesting a safer profile than traditional combinations involving pertuzumab and trastuzumab [13]. This underlines the importance of tailoring therapy based on individual patient risk and exploring alternative agents when appropriate. Overall, our study underscores the need to integrate routine cardiovascular risk assessments, personalized monitoring strategies, and cardioprotective interventions into the management of HER2-positive breast cancer. Future prospective studies with larger cohorts are warranted to confirm these findings and better characterize the long-term cardiac outcomes of HER2-targeted therapies. Further exploration of cardiac biomarkers, such as troponin and natriuretic peptides, may also improve early detection and risk stratification in this clinical setting.

## Conclusions

This retrospective study emphasizes the need to recognize cardiotoxicity risk factors in patients receiving pertuzumab and trastuzumab at the Institute of Oncology Bucharest. Identifying key demographic and clinical predictors enables healthcare providers to adopt targeted monitoring strategies and mitigate cardiac complications effectively. Future studies should focus on validating these findings prospectively and investigating the utility of biomarkers and advanced imaging techniques in predicting cardiotoxicity.

Preventing cardiotoxicity during HER2-targeted breast cancer treatments requires a multifaceted approach, including personalized risk assessments, advanced cardiac monitoring, and the use of cardioprotective medications. Incorporating biomarkers such as cardiac troponin into routine care enhances early detection and timely intervention. A key strength in managing these complex cases lies in the interdisciplinary collaboration between oncology and cardiology teams, which facilitates comprehensive care and supports optimal patient outcomes. As treatment strategies continue to evolve, fostering this collaboration remains essential to balancing therapeutic efficacy with cardiovascular safety.

A unique contribution of this study lies in its provision of real-world data from a Central-Eastern European oncology center, focusing on a patient population often underrepresented in international cardio-oncology literature. By identifying BMI as the sole independent predictor of cardiotoxicity in multivariate analysis, this study highlights the need for tailored cardiovascular risk management strategies that reflect the regional and clinical realities of resource-constrained healthcare systems.
